# Key role of a structural water molecule for the specificity of 14F7—An antitumor antibody targeting the NeuGc GM3 ganglioside

**DOI:** 10.1093/glycob/cwab076

**Published:** 2021-09-02

**Authors:** Kaare Bjerregaard-Andersen, Fana Abraha, Hedda Johannesen, Stefan Oscarson, Ernesto Moreno, Ute Krengel

**Affiliations:** Department of Chemistry, University of Oslo, NO-0315 Oslo, Norway; H. Lundbeck A/S, Valby, Denmark; School of Chemistry, University College Dublin, Belfield, Dublin 4, Ireland; Recipharm OT Chemistry, Uppsala, Sweden; Department of Chemistry, University of Oslo, NO-0315 Oslo, Norway; Department of Biosciences, University of Oslo, NO-0316 Oslo, Norway; School of Chemistry, University College Dublin, Belfield, Dublin 4, Ireland; Facultad de Ciencias Básicas, Universidad de Medellín, Medellín 050026, Colombia; Department of Chemistry, University of Oslo, NO-0315 Oslo, Norway

**Keywords:** carbohydrate-antibody interactions, N-glycolyl GM3, protein–carbohydrate interactions, water-mediated antibody specificity, water-mediated interaction, X-ray crystal structure

## Abstract

Tumor-associated glycolipids such as NeuGc GM3 are auspicious molecular targets in antineoplastic therapies and vaccine strategies. 14F7 is a monoclonal IgG1 with high clinical potential in cancer immunotherapy as it displays extraordinary specificity for NeuGc GM3, while it does not recognize the very similar, ubiquitous NeuAc GM3. Here we present the 2.3 Å crystal structure of the 14F7 antigen-binding domain (14F7 scFv) in complex with the NeuGc GM3 trisaccharide. Modeling analysis and previous mutagenesis data suggest that 14F7 may also bind to an alternative NeuGc GM3 conformation, not observed in the crystal structure. The most intriguing finding, however, was that a water molecule centrally placed in the complementarity-determining region directly mediates the specificity of 14F7 to NeuGc GM3. This has profound impact on the complexity of engineering in the binding site and provides an excellent example of the importance in understanding the water structure in antibody–antigen interactions.

## Introduction

Cancer cells differ from healthy cells by aberrant glycosylation patterns, displaying tumor-associated carbohydrate antigens (TACAs) (Dennis et al. 1987; Hakomori 2001; Pochechueva et al. 2012). Immunotherapy offers the possibility of specifically targeting TACAs with high affinity through structure-based engineering of monoclonal antibodies (Scott et al. 2000; Yu et al. 2010; Hutchins et al. 2017). The ganglioside *N*-glycolyl GM3 (NeuGc GM3) is expressed in most mammals but is absent from healthy adult human cells due to a deletion in the cytidine monophosphate-*N*-acetylneuraminic acid hydroxylase (*CMAH*) gene converting NeuAc to NeuGc (Chou et al. 1998; Irie et al. 1998). For one or more reasons (Malykh et al. 2001; Varki 2001; Yin et al. 2006; Alisson-Silva et al. 2016; Bousquet et al. 2018; Dhar et al. 2019), NeuGc GM3 is displayed to a larger extent by certain cancer cells and thus represents an attractive TACA. The monoclonal antibody (mAb) 14F7 is an IgG_1_ raised by immunizing a BALB/c mouse with NeuGc GM3 complexed with very low-density lipoproteins (Carr et al. 2000). This antibody is known for its exquisite specificity and high affinity to NeuGc GM3, determined by enzyme-linked immunosorbent assay (ELISA) to be in the low nanomolar range (Carr et al. 2000; Rojas et al. 2004; Bjerregaard-Andersen et al. 2018). 14F7 has been used to verify the presence of the NeuGc GM3 in a range of tumors including retinoblastoma (Torbidoni et al. 2015), non-small cell lung cancer (Blanco et al. 2012), colon cancer (Lahera et al. 2014), breast cancer (Carr et al. 2000; Oliva et al. 2006) and melanoma (Carr et al. 2000). Humanizing the mAb yielded 14F7hT (here referred to as 14F7 mAb), which retained its original ability to induce antibody-dependent cellular cytotoxicity in both human and murine NeuGc GM3-expressing cells (Fernández-Marrero et al. 2011; Dorvignit et al. 2019). 14F7 mAb has been reported to kill primary tumor cells by a complement-independent mechanism (Carr et al. 2002; Roque-Navarro et al. 2008); however, the details of its mode of action are unknown.

**Fig. 1:**
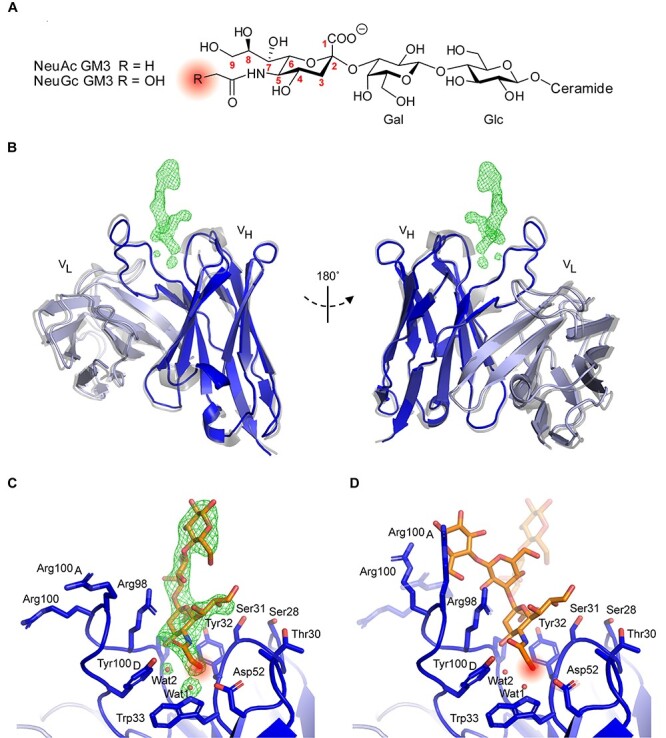
14F7 scFv complex with NeuGc GM3 trisaccharide. (**A**) Structure of ganglioside Neu5Gc and Neu5Ac GM3 with highlighted point of difference. (**B**) 14F7 scFv light (light blue) and heavy (dark blue) chains (PDB ID: 6S2I, chain A; this work). The 14F7 scFv apo-structure (PDB ID: 6FFJ (Bjerregaard-Andersen et al. 2018), chain A) is superimposed in gray. Difference electron density (*mF_o_*-*DF_c_*) for the carbohydrate ligand and key water molecules, Wat1 and Wat2, are shown at 3.0 σ (green mesh). (**C**) Structural model of 14F7scFv*–*NeuGc trisaccharide complex in synclinal conformation. Important amino acid residues and water molecules interacting with the glycan (orange) are labeled. (**D**) Alternative conformation of NeuGc GM3 trisaccharide with anticlinal glycosidic linkage between NeuGc and Gal (modeled), which buries a larger surface area on 14F7 scFv compared with the synclinal conformation observed in the crystal structure (transparent). Figure 1A was prepared with ChemDraw, Figures 1B–D with PyMOL 2.2.0.

NeuGc GM3 is composed of a ceramide tail, buried in the plasma membrane of the cell, and an exposed trisaccharide head group (Neuα2-3Galβ1-4Glcβ) featuring the sialic acid NeuGc at its tip (Labrada et al. 2018). The ability of 14F7 to effectively differentiate between the highly similar NeuGc and NeuAc epitopes is intriguing. In fact, the two glycolipids only differ by the presence of one additional oxygen atom (H to OH) present in NeuGc GM3 (Figure 1**A**). Mutational studies have highlighted key residues involved in NeuGc binding (Rojas et al. 2013), and the crystal structure of the 14F7 Fab has been solved (Krengel et al. 2004), followed more recently by the structure of a 14F7-derived single-chain variable fragment (scFv) harboring an alternative light chain (Bjerregaard-Andersen et al. 2018). While these structures revealed the architecture of 14F7’s long complementarity-determining region (CDR) H3 loop, exhibiting key residues for antigen binding, they lacked the ligand; thus, the structural basis of the discrimination between NeuGc and NeuAc GM3 remained elusive. Here we present the X-ray crystal structure of the scFv–NeuGc complex, elucidating the molecular basis for its discrimination between NeuAc and NeuGc GM3.

## Results

### Crystal structure of 14F7 scFv in complex with NeuGc GM3 trisaccharide

The structure of the 14F7 scFv in complex with the NeuGc GM3 trisaccharide was determined to 2.3 Å resolution from a single trisaccharide-soaked crystal. Data collection and refinement statistics are summarized in Table I. The crystal was obtained from the same batch of crystallization setups that earlier yielded the scFv apo-structure [Protein Data Bank (PDB) ID: 6FFJ; Bjerregaard-Andersen et al. 2018] and retained *P*2_1_ symmetry upon soaking, with similar unit cell parameters and four scFv molecules in the asymmetric unit. Two of the four scFv molecules (M1 and M2, comprised of chains A + B and C + D, respectively) were well defined by electron density in the CDR regions and could be modeled without chain breaks, whereas parts of CDR H3 could not be traced in the remaining two scFv molecules M3 and M4 (chains E + F and G + H). M1 contained additional electron density corresponding to the trisaccharide ligand (Figure 1**B**). Inspection of the ligand complex revealed that only the sialic acid component (NeuGc) of the trisaccharide interacts with the antibody (Figure 1**C**), whereas the glucose moiety extends outwards toward the solvent, where it makes contacts with residues of scFv M3 within the same crystallographic asymmetric unit. In this binding mode, the glycosidic linkage between NeuGc and Gal adopts a synclinal conformation (also referred to as “-gauche”).

**Table I:** Crystallographic data collection and refinement statistics

14F7 scFv – NeuGc complex (PDB ID: 6S2I)^a^			
**Data collection**		**Refinement**	
Beam line	ID30A-3, ESRF	Resolution (Å)	62.9–2.29 (2.34–2.29)
Wave length (Å)	0.9677	No. unique reflections	42066 (2675)
Space group	*P* 2_1_	No. reflections in test set	2151 (126)
Unit cell parameters		*R*-work / *R*-free	0.220 / 0.255
*a*, *b*, *c* (Å)	63.9 113.7 67.0	No. atoms	
*α, β, γ* (°)	90 91.2 90	Protein	7239
Solvent content (%)	51.0	Water	103
Resolution (Å)	62.9–2.29 (2.34–2.29)^b^	Ligand	44
*R*_sym_ (%)	9.7 (69.7)	*B*-factors (Å^2^)	
*R*_meas_ (%)	11.1 (76.8)	Protein	52.1
*I* / σ(*I)*	9.3 (2.1)	Water	48.6
Completeness (%)	98.8 (99.2)	Ligand	52.0
Multiplicity	4.2 (4.4)	R.m.s.d.	
CC 1/2	0.99 (0.83)	Bond lengths (Å)	0.002
Wilson *B*-factor (Å^2^)	44.0	Bond angles (°)	0.6
		Ramachandran plot	
		Favored (%)	97.3
		Allowed (%)	2.7
		Outliers (%)	0.0

^a^Data collected on a single crystal.

^b^Values in parentheses are for high-resolution shell.

**Fig. 2:**
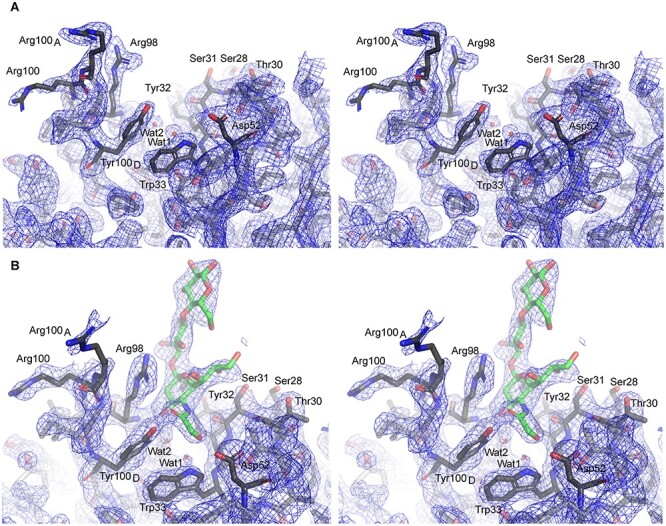
Comparison of electron density in binding sites of apo and ligand-bound scFv. Stereo images of the ganglioside binding sites of apo (**A**) and ligand-bound (**B**) 14F7 scFv, showing unbiased electron density from *2mF*_*o*_*-DF*_*c*_ composite omit maps at 1.0 σ (blue mesh), mapped to the binding pockets, including key water molecules Wat1 and Wat2. The ligand-bound (**B**) structure displays slightly weaker electron density, but the binding site architecture remains well defined, including the positions of Wat1 and Wat2. The figure was prepared with PyMOL 2.0.0.

Overall, the structure of the scFv–NeuGc GM3 trisaccharide complex is highly similar to the previously published scFv apo-structure (Bjerregaard-Andersen et al. 2018), with an average root mean square difference (r.m.s.d.) of 0.6 Å for Cα atoms indicating very little structural change upon binding (Figure 1**B**). Also, the side chain conformations of amino acid residues in proximity of the saccharide binding site are very similar between the scFv complex and the apo-structure. Tyr32 and Tyr100_D_, both in direct contact with the ligand through H-bonds, readjust by approximately 1 Å to accommodate binding. Most noticeably, Arg98 adopts a new conformation upon ligand binding, where it stacks against the sialic acid residue of the NeuGc GM3 trisaccharide (Figures 1**C** and 2). The antigen-binding site also contains two water molecules binding the ligand.

2*mF_o_*-*DF_c_* composite omit maps of both the apo (PDB ID: 6FFJ; Bjerregaard-Andersen et al. 2018) and the ligand-bound scFv structures (PDB ID: 6S2I; this work) show that the 14F7 binding pocket architecture is well defined by unbiased electron density, including the positions of water molecules Wat1 and Wat2 (Figure 2**A** and **B**). Only Arg100 and Arg100_A_ remain slightly less ordered owing to their dynamic nature, with the ligand adopting synclinal conformation.

### Structural basis for 14F7 discrimination between NeuGc and NeuAc GM3

**Fig. 3:**
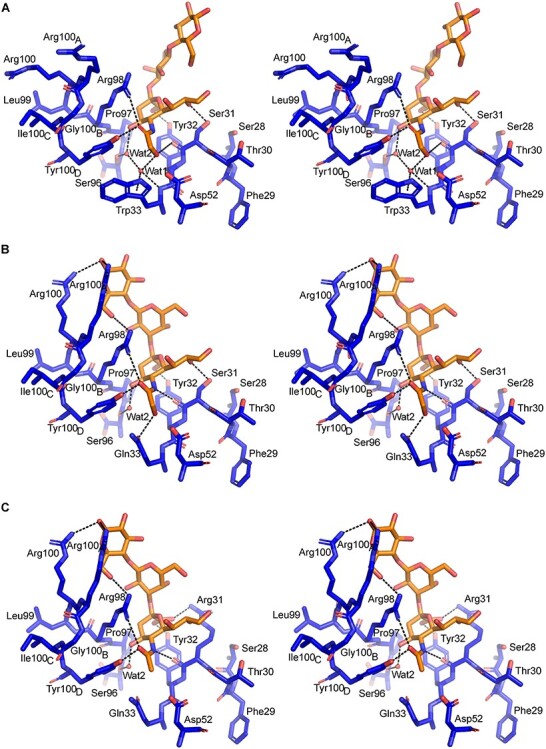
Stereo pictures showing the specificity of Trp33, W33Q and W33Q/S31R 14F7 variants in complex with NeuGc or NeuAc. (**A**) Crystal structure of 14F7 Trp33 (blue) bound to NeuGc (orange) in its experimentally determined conformation (PDB ID: 6S2I, chain A; this work). (**B**) Model of 14F7 W33Q variant, with NeuGc in the in silico-optimized anticlinal conformation. (**C**) Model of the cross-reactive 14F7 S31R/W33Q variant, with NeuAc in the anticlinal conformation. The figure was prepared with PyMOL 2.0.0.

The interactions between 14F7 and the NeuGc GM3 trisaccharide are shown in Figure 3**A** and listed in Table II. 14F7 has repeatedly been shown to strongly differentiate between NeuGc and NeuAc GM3 in vitro, e.g., probed by ELISA (Carr et al. 2000; Bjerregaard-Andersen et al. 2018). Therefore, the key determinant for discrimination must be found in the trisaccharide head group, where the only difference is the presence of an additional hydroxyl group in the *N*-glycolyl moiety of the sialic acid. Intriguingly, the *N*-glycolyl hydroxyl group does not itself provide any direct interaction with the scFv, except for a backbone interaction with Tyr32 but manifests its presence through a water molecule (Wat1; Figures 1**C**, 2**B** and 3**A**). Wat1 is part of a hydrated pocket coordinated by Trp33 and is also present in the 14F7 scFv apo-structure (PDB ID: 6FFJ; Bjerregaard-Andersen et al. 2018); thus, it may be regarded as an extension of CDR H1. Wat1 not only interacts with the *N*-glycolyl hydroxyl group of NeuGc, but also with its 4-OH group, via a second water molecule (Wat2), which binds to the backbone oxygen of Ser96 (Figure 3**A**). On the protein side, Wat1 establishes an H-bond with the backbone NH of Trp33 and a weaker, out-of-plane H-bond with the aromatic π face of its indole pyrrole ring (Figure 3**A**). Mutagenesis of Trp33 reveals that specificity is only maintained when this residue is exchanged by another aromatic residue, i.e*.*, Phe and Tyr (Rojas et al. 2013). Especially the possible replacement by Phe emphasizes the importance of the aromatic interaction with Wat1. This trisaccharide–water complex, unable to form with NeuAc, places itself like a cassette into the bottom of the binding pocket formed by the backbone and side chains of Ser31, Tyr32, Trp33, Asp52, Pro97, Arg98 and Tyr100_D_ (shown in Figure 3**A**).

**Table II:** Protein–carbohydrate interactions

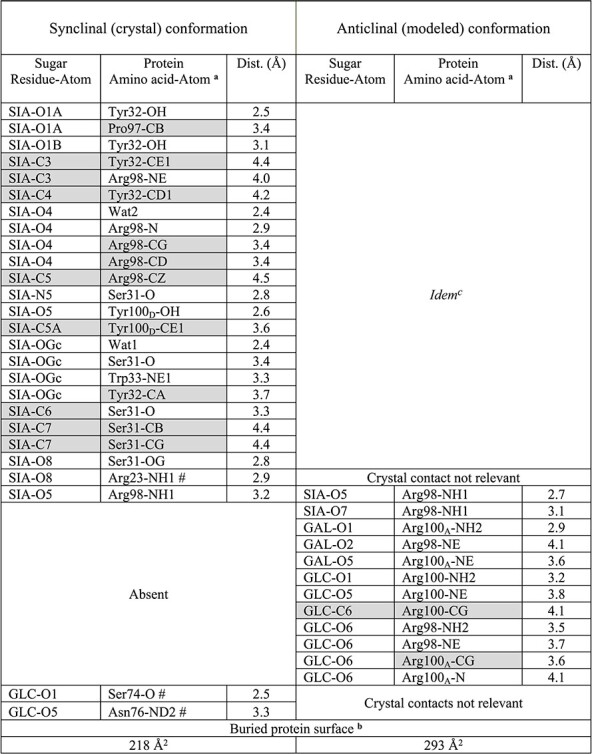

Atomic contacts between sugar and protein/water atoms for the synclinal (crystal) and anticlinal (modeled) conformations, defined by the sialic acid–galacto*se glycosidic linkage.* Packing contacts with distances up to 4.5 Å were also included in the table. Contacting carbon atoms are shaded in light gray.

^a^Amino acid atom names follow PDB conventions, # marks a neighboring molecule in the crystal.

^b^In these calculations, the two buried waters were considered part of the protein. The contribution of the neighboring chain (#), due to crystal packing, was not included in the calculations. ^c^*idem* is a Latin term meaning “the same”.

### Alternative trisaccharide binding mode

In the crystal, NeuGc GM3 adopts synclinal torsion angles between NeuGc and Gal. In solution, a common alternative conformation of the NeuGc GM3 trisaccharide has an anticlinal glycosidic linkage (Siebert et al. 1992), which in the crystal is hindered by crystal contacts. Since it is likely that both carbohydrate conformations are accessible in solution, we modeled an alternative binding mode for the trisaccharide, where NeuGc remained exactly as in the crystal structure, but the two torsion angles of its glycosidic linkage with galactose adopt the anticlinal conformation (Figure 1**D**). We found that this binding geometry brings additional favorable contacts between the trisaccharide and CDR-H3, including interactions between both Arg100 and Arg100A with the trisaccharide glucose residue (Table II). It also increases the buried surface area by more than one third, from 218 Å^2^ to 293 Å^2^. Furthermore, in this binding mode, Arg98 becomes more tightly packed against the trisaccharide (Figures 1**D**, 3**B** and 3**C**). This is in good agreement with mutagenesis data showing the critical role of this amino acid, which did not tolerate any substitution (Rojas et al. 2013).

### Models of 14F7 variants explore functional mapping data

In previous work, we used phage display to perform extensive mutagenesis studies on the 14F7 heavy chain CDRs (Rojas et al. 2013). These studies identified several positions in CDRs H1 and H3 as important for recognizing NeuGc GM3, e.g*.*, Trp33, Asp52, Arg98, Arg100, Arg100_A_ and Tyr100_D_. In addition, we found that several single-residue substitutions, yielding, e.g., S28R, T30R, S31R and W33Q conferred different levels of cross-reactivity to the antibody, and some double or triple combinations even raised the affinity to NeuAc GM3 to the same level as for NeuGc GM3 (Rojas et al. 2013). Here we modeled one of these variants (W33Q) in complex with NeuGc GM3 (Figure 3**B**) and another (S31R/W33Q) in complex with NeuAc GM3 (Figure 3**C**), in order to interpret the mutagenesis data. The introduction of an arginine residue in the antigen-binding site is likely to yield a salt bridge with the sialic acid carboxylate. Gln33 (as in W33Q) probably interacts directly with the *N*-glycolyl OH of NeuGc GM3, replacing Wat1 (Figure 3**B**).

## Discussion

Gangliosides are sialic-acid containing glycosphingolipids present in the plasma membranes of all vertebrates. They are functionally important and are known to modulate cellular signaling (Bremer et al. 1984; Hakomori 2002; Klokk et al. 2016; Cheng and Smith 2019). Despite decades of studies, the structure and function of these cell surface antigens remain to be fully appreciated, and only few anti-ganglioside antibodies, such as 14F7, have been raised (Krengel and Bousquet 2014). We previously solved the crystal structure of the 14F7 Fab and generated a computer model of its complex with the NeuGc GM3 trisaccharide (Krengel et al. 2004). However, the crystals were difficult to reproduce, and we had limited supplies of the expensive trisaccharide for co-crystallization. After recent mutagenesis data (Rojas et al. 2013) revealed shortcomings of the earlier computer model, we designed a scFv construct of 14F7 for detailed structural analysis (Bjerregaard-Andersen et al. 2018).

The new experimental data of the carbohydrate complex explain how 14F7 distinguishes the very small chemical difference between the gangliosides NeuGc and NeuAc GM3 (Carr et al. 2000; Bjerregaard-Andersen et al. 2018), and even more remarkably, we have now discovered that it does so indirectly, through a water molecule. NeuGc GM3 engages in two water-mediated interactions with Trp33, one with its main chain amine and one with the π-system of the indole side chain (both through Wat1; Figure 3**A**). Such an interaction is weaker than an ordinary hydrogen bond (Gierszal et al. 2011); however, the importance of this interaction is highlighted by the fact that substitution of Trp33 with Phe or Tyr retains specificity, whereas nonaromatic residues abolish binding or allow cross-reactivity with NeuAc GM3 (Rojas et al. 2013). H-bonds commonly mediate specificity in antibody–antigen recognition through direct contact between paratope and epitope side chains (Peng et al. 2014). In the case of 14F7, Wat1 is already present in the protein apo-structure (PDB ID: 6FFJ; Bjerregaard-Andersen et al. 2018) (Figure 2**A**). A thorough analysis of water–tryptophan interactions indicates that the six-membered ring of the indole side chain favors π-OH interaction, whereas the five-membered pyrrole ring favors π-lone pair interaction (Durec et al. 2018). The latter appears to be the case for Wat1, thus positioning it as an H-bond donor for the *N*-glycolyl group of NeuGc GM3. While it is well-known that the hydration shell is important for protein structure and function (Levy and Onuchic 2006; Bellissent-Funel et al. 2016), including the recognition of carbohydrates (Weis and Drickamer 1996; Janin 1999; Holmner et al. 2004) and antibody–antigen interactions (Braden et al. 1995; Cohen et al. 2005; Horita et al. 2016; Marino et al. 2016), the complexity of antibody engineering is highlighted by our finding of this indirect, water-mediated specificity.

### Selectivity vs. cross-reactivity

NeuGc is bound to the bottom of a cleft formed by the variable heavy chain of 14F7 (Figure 3**A**), which is separated from the variable light chain through the long CDR H3 loop. The predicted NeuGc recognition site has previously been functionally mapped by a combinatorial phage display strategy using an alternative format of 14F7 scFv (Rojas et al. 2013). The study revealed that substitution of Trp33 in combination with residues 28, 30 or 31 could yield cross-reactive 14F7 variants (e.g., S28R/S30R/W33Q, S31R/W33Q and S28R/S31R, and to a lesser extent by single amino acid substitutions) (Rojas et al. 2013). Therefore cross-reactivity is likely mediated through direct interactions with the sialic acid residue, in particular by a salt-bridge to the negatively charged carboxylate group found in both NeuGc and NeuAc GM3. Substituting Trp33 as in 14F7 W33Q likely leads to the replacement of Wat1 by the glutamine side chain amide, which can interact directly with the *N*-glycolyl OH of NeuGc GM3 (Figure 3**B**). This mutation alone decreased NeuGc GM3 binding but promoted a weak interaction to the NeuAc variant of GM3 (Rojas et al. 2013).

To further explore the mutagenesis data, we modeled the 14F7 S31R/W33Q variant in complex with NeuAc GM3. Substitution of Ser31 with Arg (S31R) trades an H-bond to one of the NeuGc glycerol hydroxyls for a charge interaction of the guanidinium moiety with the sialic acid carboxyl group found in both NeuGc and NeuAc GM3 (Figure 3**C**), thus conferring some cross-reactivity to the antibody. Arginine substitutions of Ser28 (S28R) or Thr30 (T30R) likely elicit similar effects. Interestingly, in spite of this additional interaction, substituting Ser31 for Arg, either alone or combined with other amino acid substitutions, hardly increased the affinity for NeuGc GM3 (Rojas et al. 2013).

Tyr100_D_ directly binds to the *N*-glycolyl group of NeuGc and likely contributes to maintaining the architecture of the binding pocket, since substitution with nonaromatic amino acids abolishes binding. While Y100_D_W maintains specificity to NeuGc, Y100_D_F yielded cross-reactivity to NeuAc (Rojas et al. 2013). The inability of Phe to H-bond to NeuGc/Ac is expected to weaken binding to NeuGc such that it may become indistinguishable from NeuAc. Alternatively, the cross-reactivity could be mediated through an alternative conformation of NeuAc in the binding pocket.

Asp52 faces the *N*-glycolyl group of NeuGc, but keeps a distance of approximately 4 Å to the ligand. Even though Asp52 does not bind directly to NeuGc, it appears to be important for maintaining the binding site architecture through hydrogen bonding to the Trp33 indole nitrogen as well as to the CDR H2 backbone. Moreover, Asp52 helps to position Wat1 (and its interaction network via Wat2 to NeuGc OH4) via Trp33 binding. Substitutions D52A/E/N/S/T retain binding to NeuGc but allow cross-reactivity to NeuAc, whereas replacement with bulky or hydrophobic residues (C/F/H/K/P/R/V/Y) abolishes binding (Rojas et al. 2013), likely by disturbing the binding site architecture.

Although it may seem counterintuitive that NeuAc could bind to a polar pocket, a polar environment is not unprecedented for NeuAc. For example, cross-reactive rotaviruses that recognize both NeuAc and NeuGc GM3 have been shown to display similar polar, water-containing pockets to accommodate the acetyl or glycolyl groups of their glycan receptors (Yu et al. 2011). Favorable interactions elsewhere, e.g., with the sialic acid carboxylate or glycerol chain, may compensate for less favorable interactions of the *N*-acetyl group. In fact, it is likely that selectivity of NeuGc over NeuAc GM3 requires a fine balance of interactions, and that too tight binding of the sialic acid residue may prevent selectivity and would tip the balance toward cross-reactivity toward NeuGc and NeuAc GM3. Further engineering to obtain high affinity without cross-reactivity should be centered on replacing the water-mediated interaction of the *N*-glycolyl hydroxyl with a direct amino acid interaction, such as that of the W33Q mutation. In fact, W33Q may be a viable starting point for engineering of 14F7 NeuGc preference and affinity.

### Glycan conformation and antibody recognition

In the crystal structure of the scFv–saccharide complex (PDB ID: 6S2I; this work), the saccharide adopts a synclinal conformation (Figure 1**C**), and the only interaction with 14F7 is via the sialic acid (Figure 3**A**). A common alternative conformation (Siebert et al. 1992; DeMarco and Woods 2009), with anticlinal glycosidic linkage, is hindered by crystal contacts. In a biological context (and in solution), the saccharide is likely free to adopt both conformations, also the anticlinal conformation (Figure 1**D**), which provides a larger contact surface with the antibody (293 vs. 218 Å^2^) (Table II). Dynamic binding in two alternative conformations may in fact provide an entropic advantage. In both conformations, the glycosidic linkage between NeuGc and Gal places the key CDR H3 residue Arg98 in a central position for interaction with the NeuGc GM3 trisaccharide (Figure 3**A** and 3**B**), explaining why any substitution of this residue renders it incompatible with binding. In anticlinal conformation, Arg98 can additionally interact with the glucose moiety of NeuGc GM3 through H-bonds. This is also true for Arg100 and Arg100_A_, which are located at the tip of CDR-H3. Moreover, the arginine residues exposed on CDR H3 create a strongly positively charged surface patch that will likely also interact with other components of the plasma membrane. The observation that these residues, in general, can be exchanged while maintaining a positive charge (Rojas et al. 2013), indicates nonspecific interactions with the membrane through negative charges found in the proximity of the target antigen, such as other phospholipids, gangliosides or proteins. It will be exciting to explore how 14F7 recognizes NeuGc GM3 in its membrane environment.

## Methods

### Synthesis of NeuGc trisaccharide

The NeuGc GM3 trisaccharide was synthesized through an IBr/AgOTf-promoted glycosylation of a benzylated lactose acceptor with a NeuGc thioglycoside donor, followed by global deprotection of the obtained trisaccharide (Bjerregaard-Andersen et al. 2018).

### Expression and purification of 14F7-derived scFv

The 14F7 scFv was produced by a variation of a protocol described by Bjerregaard-Andersen et al. (2018). Compared with the original 14F7 mAb, this construct contains an alternative light chain identified by Rojas et al. (2004). The linker was chosen on the basis of a vector system established for expression of single-chain T-cell receptors and scFvs in *Escherichia coli* (Løset et al. 2005; Gunnarsen et al. 2010)*.* Briefly, the scFv was expressed in *E. coli* by a pFKPEN vector-based system. The vector encodes a *pelB* leader sequence, thus promoting the translocation of the protein to the periplasm. Purification included limited lysis of the *E. coli* outer membrane to release the mature scFv and subsequent purification by protein L affinity chromatography and size exclusion chromatography to reach a highly pure and homogenous preparation for crystallization and binding experiments.

### Crystallization of the 14F7 scFv and soaking with NeuGc trisaccharide

Crystallization of the 14F7 scFv was performed by Bjerregaard-Andersen et al. (2018). Crystals of good diffraction quality were obtained from the Morpheus screen (Hampton Research, USA) after seeding with small crystals from initial hits. Remaining crystals from the D12 condition (12.5% w/v PEG 1000, 12.5% w/v PEG 3350, 12.5% v/v MPD, 0.02 M 1,6-hexandiol, 0.02 M 1-butanol, 0.02 M(*RS*)-1,2-propanediol, 0.02 M 2-propanol, 0.02 M 1,4-butanediol, 0.02 M 1,3-propanediol, 0.1 M Bicine/Tris base pH 8.5), used for determination of the 14F7 scFv apo-structure (Bjerregaard-Andersen et al. 2018), were soaked by the addition of the synthesized NeuGc trisaccharide in powder form. The crystals were incubated for 1 h before flash cooling in liquid nitrogen and stored for diffraction experiments.

### Data collection and structure determination

Diffraction data extending to 2.3 Å were collected at the ID30A-3 beam line at the European Synchrotron Radiation Facility (ESRF), Grenoble, France. X-ray data were auto-processed at the ESRF by the *EDNA* pipeline (Incardona et al. 2009). The structure was phased by molecular replacement with the *PHENIX* crystallographic software package (Adams et al. 2010), using the 14F7 scFv apo-structure (PDB ID: 6FFJ; Bjerregaard-Andersen et al. 2018) as search model and refined in alternating cycles of manual model building and refinement with *PHENIX* (Adams et al. 2010) and *Coot* (Emsley et al. 2010). Water molecules were built in at late stages of the refinement, initially using the automated water picking routine of *PHENIX* (Adams et al. 2010). These sites were then inspected individually and assessed for removal in case of electron density sigma level >1.10 e/Å^3^ or bond distances >3.5 Å or <2.2 Å. Likewise, missing water molecules were added manually. The phased map revealed additional electron density in one of the four scFv molecules in the asymmetric unit, which was modeled as NeuGc GM3 trisaccharide. The trisaccharide ligand was built using *eLBOW* (Moriarty et al. 2009) and modeled into the electron density of the binding pocket at final stages of structure building and adjusting occupancy by matching ligand *B*-factors to interacting protein residues. An OMIT difference density map was made by removing the trisaccharide ligand from the final model, followed by five refinement cycles using *PHENIX* (Adams et al. 2010). Likewise, composite OMIT maps were generated using *PHENIX* (Adams et al. 2010). The final model was deposited in the PDB with accession code 6S2I.

### Modeling

The program *VMD* (Humphrey et al. 1996) was used to for visualization and analysis as well as for molecular modeling. The two amino acid substitutions in the heavy variable domain—S31R and W33Q—were made using the Mutator plugin implemented in *VMD*. Side chain conformations were modeled using the Molefacture plugin. The same tool was used to model the anticlinal conformation of the GM3 trisaccharide, keeping the sialic acid in its crystal position and modifying only the two torsion angles of its glycosidic linkage with galactose.

## Author contributions

U.K. conceived the study. F.A. synthesized the trisaccharide, supervised by S.O. H.J. expressed and purified the constructs, supervised by U.K. K.B.-A. was in charge of the crystallography, with U.K. validating the crystal structure, and E.M. performed the modeling studies. K.B.-A. wrote the first draft of the manuscript, which was revised in tight collaboration with H.J., E.M. and U.K., and approved by all authors.

## Abbreviations

CDR, complementarity-determining region; CMAH, cytidine monophosphate-*N*-acetylneuraminic acid hydroxylase; ELISA, enzyme-linked immunosorbent assay; ESRF, European Synchrotron Radiation Facility; Gal, galactose; Glc, glucose; mAb, monoclonal antibody; Neu, neuraminic/sialic acid; NeuAc, *N*-acetyl (Neu5Ac); NeuGc, *N*-glycolyl (Neu5Gc); PDB, Protein Data Bank; r.m.s.d., root mean square difference/deviation; scFv, single-chain variable fragment; TACA, tumor-associated carbohydrate antigen.
